# Intravenous oxygen administration in a rat model of hypoxia

**DOI:** 10.1186/2197-425X-3-S1-A575

**Published:** 2015-10-01

**Authors:** E Damiani, A Donati, M Singer

**Affiliations:** Biomedical Sciences and Public Health, Università Politecnica delle Marche, Ancona, Italy; University College London, Bloomsbury Institute of Intensive Care Medicine, London, United Kingdom

## Introduction

Hypoxemia reduces tissue oxygen delivery, thus compromising cell metabolism and organ function. Supplemental oxygen at high concentrations may prove ineffective and issues relating to hyperoxia, barotrauma, mechanical ventilation and extracorporeal oxygenation are well documented [1, 2]. A century ago, Tunnicliffe et al reported rapid and safe relief of cyanosis in patients by administration of intravenous oxygen gas [3]. This re-discovered route warrants re-exploration.

## Objectives

To test the safety and efficacy of intravenous administration of oxygen either as a pure gas or dissolved in Ringer's Lactate (RL) solution saturated to 100%.

## Methods

Under isoflurane anesthesia, male Wistar rats (about 300 g bw) underwent arterial and central venous cannulation, tracheotomy, bladder cannulation and placement of tissue PO_2_ probes (Oxford Optronix, Oxford, UK) in leg muscle and liver. Hypoxia was induced by breathing a hypoxic gas mix (FiO_2_ 0.1). At 60 minutes, a continuous iv infusion of pure O_2_ gas (2 mL/kg/h) or oxygenated RL (10 mL/kg/h) was begun. An equal volume of normal RL was given to controls. Echocardiography, arterial blood gas analysis, mean arterial pressure (MAP), urine output, muscle and liver tPO_2_ were measured at baseline and at hourly intervals for 4 hours.

## Results

**Figure 1:**
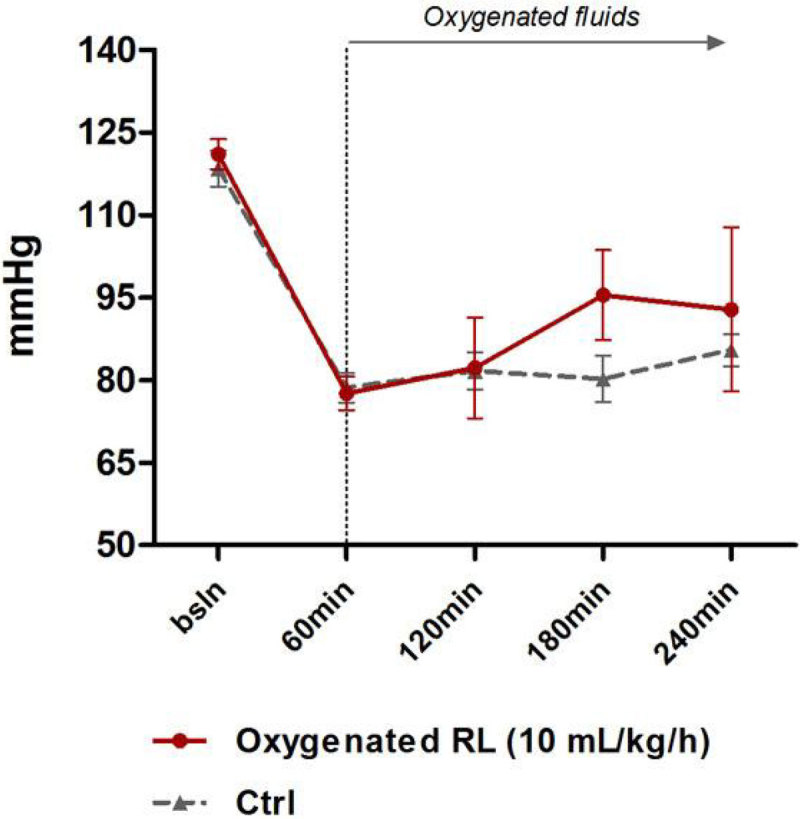
**Mean arterial pressure.**

**Figure 2:**
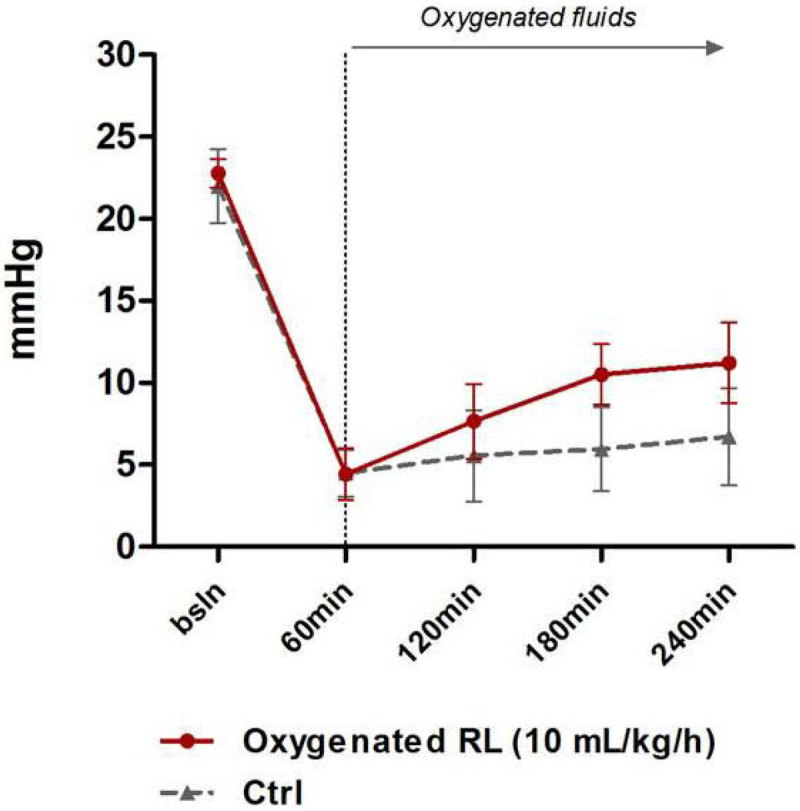
**tPO**_**2**_ **(liver).**

**Figure 3:**
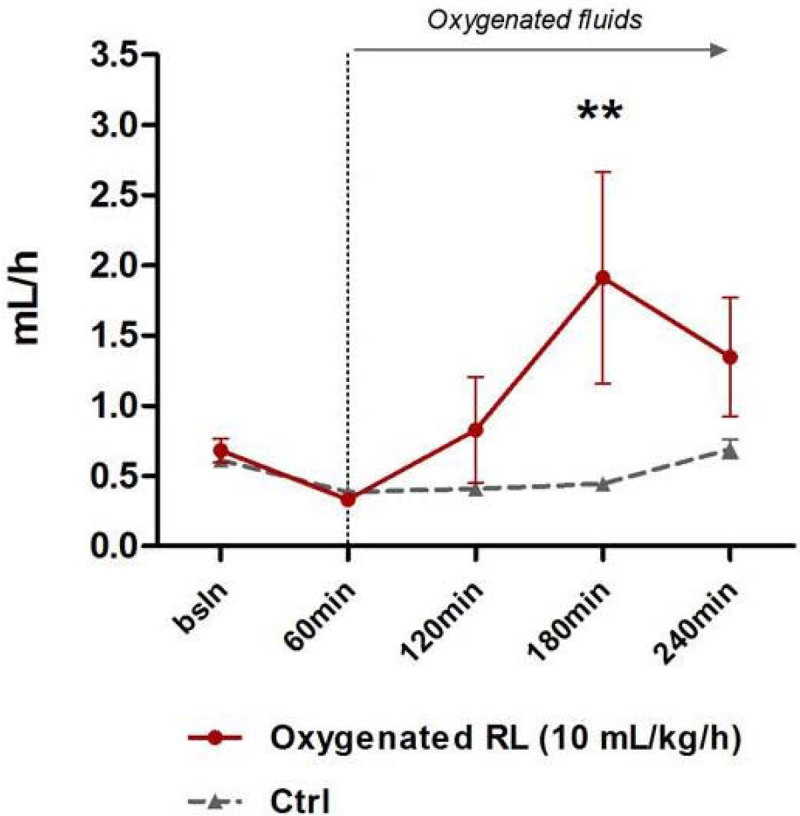
**Urine output.**

Infusion of pure O_2_ gas caused early death due to pulmonary embolism so this technique was abandoned. Administration of oxygenated RL (PO_2_ of solution at end-experiment = 87.5 ± 1.7 kPa) was however safe but did not produce any significant increase in PaO_2_ or SaO_2_, in comparison to controls. However, O_2_ delivery, MAP (Figure 1) and liver PO_2_ (Figure 2) (but not muscle PO_2_) rose progressively with oxygenated RL with urine output increasing to supranormal values (Figure 3).

## Conclusions

In this rat model of hypoxia, the intravenous infusion of oxygenated RL was safe. While it did not produce any increase in arterial or muscle oxygenation, it did appear to impact on the splanchnic circulation, increasing liver PO_2_ and urine output.
